# Nanoparticles in HIV treatment for improved drug delivery, clinical translation, and future direction

**DOI:** 10.1186/s11671-025-04427-z

**Published:** 2026-01-08

**Authors:** Swase Dominic Terkimbi, Reuben Samson Dangana, Solomon Adomi Mbina, Ugwu Okechukwu Paul-Chima, Patrick Maduabuchi Aja, Regan Mujinya

**Affiliations:** 1 Department of Biochemistry, Kampala International University, Ishaka, Uganda; 2 Discipline of Genetics, School of Life Sciences, University of KwaZulu-Natal (Westville), Durban, South Africa; 3 Department of Physiology, Kampala International University, Ishaka, Uganda; 4 Department of Physiology, Equator University of Science and Technology, Masaka, Uganda; 5 Department of Publication and Extension, Kampala International University, Kampala, Uganda; 6 Department of Pharmacy, Facuty of Health Sciences, Victoria University, Kampala , Uganda

**Keywords:** Nanoparticles, Control release, Viral reservoirs, Biodistribution, And pharmacokinetics

## Abstract

**Abstract:**

HIV remains a major global health challenge with antiretroviral therapy (ART) effectively suppressing viral replication. However traditional ART does not eliminate viral reservoirs and is limited by systemic toxicity, long-term adherence burdens, and incomplete tissue penetration. These limitations highlight an important scientific problem in the inability of conventional ART to achieve durable remission or cure. Nanoparticle-mediated drug delivery systems have emerged as a transformative approach to address these limitations by improving drug solubility, stability, and targeted delivery to infected cells and viral sanctuaries such as the brain, lymphoid organs, and gastrointestinal mucosa. Different nanocarrier platforms including liposomes, polymeric nanoparticles, dendrimers, and lipid-based vesicles enable both passive and active targeting strategies. Functionalization with ligands such as antibodies, peptides, aptamers, and sugar moieties enhance cellular uptake, reduces off-target effects, and optimizes pharmacokinetics and biodistribution. Controlled-release formulations extend drug half-life and reduce dosing frequency, supporting long-acting regimens. Beyond drug delivery, nanoparticles also facilitate immunomodulatory therapies, therapeutic vaccines, and advanced gene-editing technologies such as CRISPR–Cas9. The convergence of nanotechnology, mRNA platforms, and artificial intelligence-driven drug development represents a paradigm shift toward individualized and precision HIV treatment. Despite these advances, significant translational challenges remain, including nanotoxicity, long-term safety, large-scale GMP manufacturing, regulatory barriers, and cost-effectiveness. Addressing these barriers is essential to unlock the full potential of nanoparticle-based strategies and translate them into equitable and sustainable clinical solutions.

**Graphical abstract:**

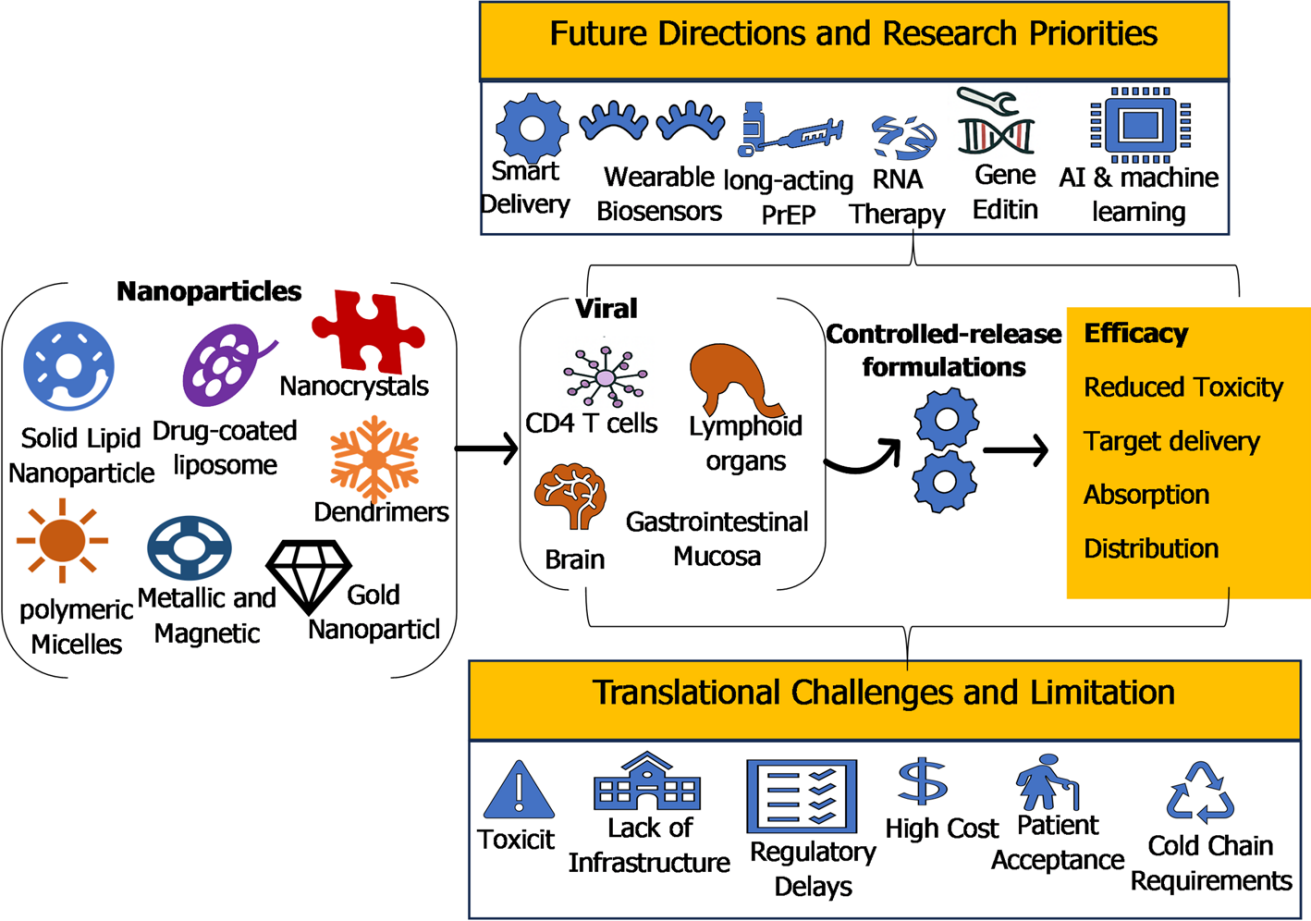

## Highlights

This review synthesizes evidence on nanoparticle-mediated antiretroviral delivery, focusing on pharmacokinetics, biodistribution, and viral reservoir targeting.

Lipid- and polymer-based nanoparticles demonstrate measurable improvements in lymphoid tissue penetration and sustained plasma concentrations in preclinical models.

Long-acting nanosuspensions of cabotegravir and rilpivirine provide clinical proof-of-concept for bi-monthly injectable HIV therapy.

Emerging nanoplatforms enable integration with RNA therapeutics, immunomodulators, and CRISPR-based gene editing for future personalized interventions.

Key translational challenges include nanotoxicity, GMP-scale manufacturing, regulatory frameworks, and cost-effectiveness, particularly in resource-limited settings.

## Introduction

Nanoparticle-mediated drug delivery systems represent a paradigm shift in biomedical therapeutics, offering innovative solutions to the challenges and limitations of conventional ART [1]. Traditional ART have proven to effective but limited by poor bioavailability, rapid clearance requiring lifelong daily dosing, systemic toxicity, and poor patient adherence. This compromise long-term viral suppression and increase the emergence of resistance to treatment. These limitations are further compounded by pharmacogenomic variability, where patient-specific genetic differences in drug-metabolizing enzymes (e.g., CYP450 isoforms) influence ART pharmacokinetics and toxicity profiles. Likewise, variations in drug transporters such as P-glycoprotein and immune response genes further modulate drug response and treatment outcomes [2]. Such inter-individual variability contributes to unequal therapeutic efficacy, adverse drug reactions, and disparities in virologic suppression, particularly in genetically diverse populations [3]. Nanoparticles provide a promising strategy to overcome these challenges by improving drug stability, enhancing solubility, bypassing first-pass metabolism, and enabling controlled or sustained release. Importantly, nanocarrier systems can minimize the influence of pharmacogenomic differences by delivering consistent therapeutic concentrations, reducing reliance on hepatic metabolism pathways affected by genetic polymorphisms, and directly targeting HIV-infected cells or reservoirs [4].

Nanoparticles typically ranging from 1 to 100 nanometers are made up of different physicochemical properties, which enable them to control and release therapeutic agents directly into diseased tissues or HIV infected cells. These systems have demonstrated significant success across different medical sectors, including oncology, cardiology, and infectious disease management [5]. The use of nanoparticles in HIV therapeutics dates back to the 1990s, with early investigations into liposomes and polymeric nanoparticles. This system was designed to improve drug stability and controlled release. Progress continued into the early 2000s with the development of metallic nanoparticles and dendrimers, enhancing targeting efficiency and reducing toxicity profiles. A significant breakthrough took place 2010s with the introduction of long-acting injectable nanoparticle formulations, such as nanocrystal-based cabotegravir and rilpivirine, which revolutionised ART by improving treatment adherence and long-term viral suppression. The introduction of modern nanoparticle platforms such as liposomes, dendrimers, solid lipid nanoparticles, and biodegradable polymers like poly (lactic-co-glycolic acid) (PLGA) offers several advantages in ART delivery [6]. These include enhanced protection of drugs from enzymatic degradation, bypassing first-pass hepatic metabolism, improved solubility of hydrophobic agents, and prolonged systemic circulation [7]. Nanoparticle-based drug delivery has been widely studied in HIV therapy, yet most studies focus on laboratory advances without addressing clinical translation, regulatory challenges, and real-world applicability in resource-limited settings. This review bridges that gap by synthesizing evidence from clinical trials of long-acting formulations such as cabotegravir and rilpivirine, while examining regulatory progress, implementation challenges, and future directions. It also looks at emerging applications, including nanocarriers for gene-editing tools, highlighting their potential for improving adherence, safety, and equitable access in low-resource contexts.

## Methodology

**Fig. 1:**
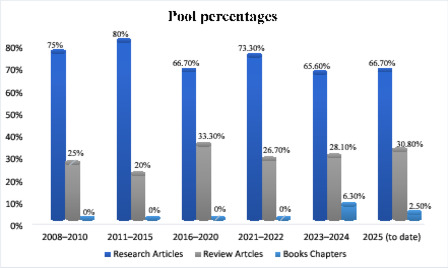
Distribution of Publications on Nanoparticle-Mediated HIV Drug Delivery (Scopus Database, up to 2025). The Bibliometric screening of the included revealed a progressive increase in Scopus-indexed nanomedicine and HIV publications, particularly between 2023–2025, reflecting expanding global interest in nano-ART and long-acting delivery strategies. Research articles dominated (68.8%), followed by reviews (28.4%) and book Chaps. (2.8%), indicating both active experimental work and growing synthesis of prior knowledge. The rise in reviews and book chapters after 2023 highlight consolidation and maturity in the field, aligning with the emergence of AI-based formulation design and clinically approved long-acting nano-ART

This study employed a bibliometric analysis using the Scopus database, which was selected because of its wide coverage of peer-reviewed journals, books, and scientific reviews. A systematic search was conducted using combinations of keywords such as nanoparticles and HIV, nanomedicine and antiretroviral therapy, liposomes and HIV, and nanocarriers and drug delivery. The search was carried out without year restrictions to capture all relevant publications up to 2025. Only peer-reviewed research articles, review articles, and books or book chapters were considered for inclusion, while non-English publications, editorials, opinion pieces, conference abstracts, and documents lacking complete bibliographic information were excluded. Metadata including document type, author, year, journal, and DOI were extracted from the included records. Each publication was classified into one of three categories such as research article, review article, or book/book chapter. Descriptive statistics were applied to determine the distribution of publications across these categories. Results were expressed in percentages with graphical visualizations trend is presented in Fig. 1 was used to highlight the relative contribution of each category and to illustrate publication trends.

## Classification of nano-formulations for HIV drug delivery

**Fig. 2:**
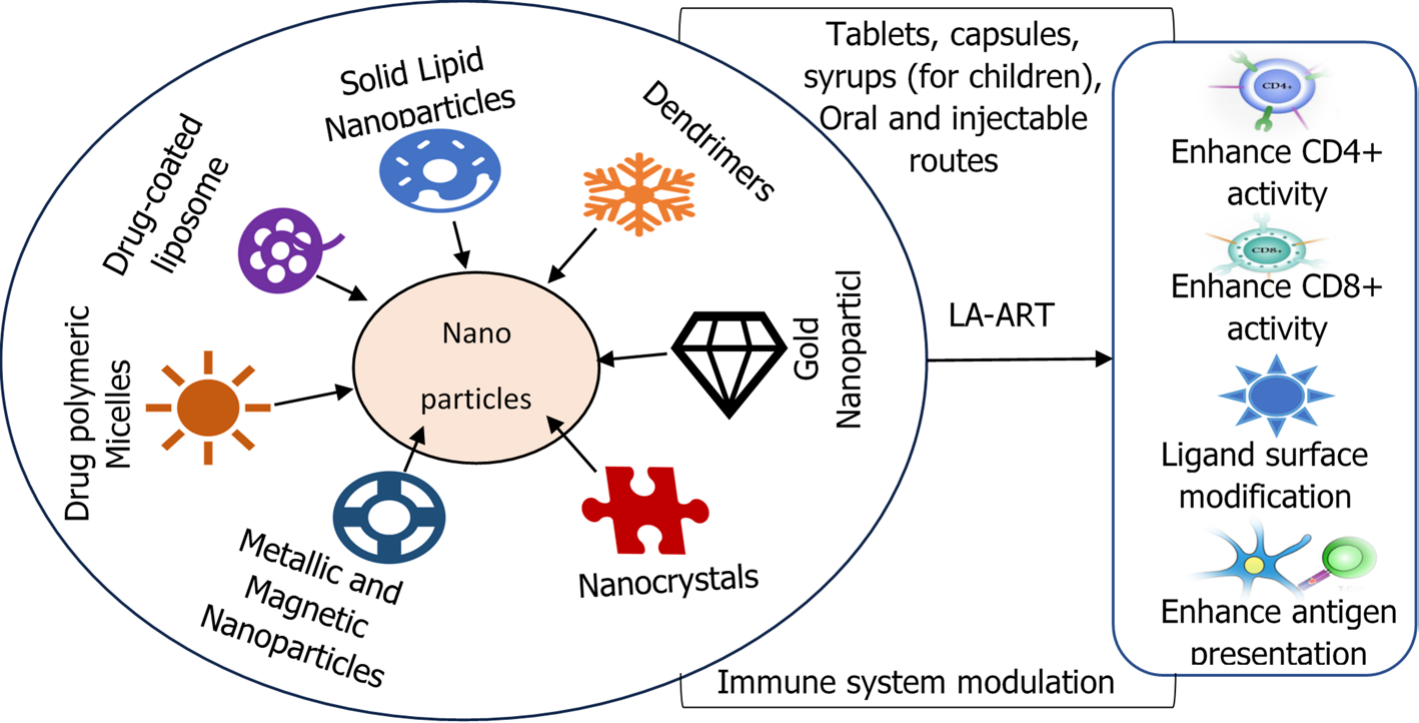
Nanoparticle-Mediated Targeted Drug Delivery in HIV Treatment. The central panel depicts different classes of nanoparticles including liposomes, polymeric nanoparticles, dendrimers, solid lipid nanoparticles, nanocrystals, metallic nanoparticles, and hybrid carriers highlighting their structural versatility. The right panel illustrates their ability to be functionalized for selective targeting of key HIV reservoir cells such as CD4⁺ T cells, macrophages, dendritic cells, and latently infected T cells

Nanoparticle-based drug delivery systems can be classified into vesicular systems, lipidic systems, polymeric systems, inorganic systems, and hybrid/novel systems as depicted by Fig. 2. Each category as discussed in subsection is characterized by different physicochemical properties, mechanisms of drug encapsulation, and biological interactions that influence HIV therapy.

### Vesicular systems

Vesicular systems are made up of amphiphilic architecture including bilayer rigidity, phase transition temperature (Tm), and membrane elasticity. These features enable loading of drug-loading capacity (both hydrophilic and hydrophobic), stability, biodistribution, and intracellular delivery of antiretroviral (ARV) agents [8]. Bilayer rigidity and Tm are central to vesicle performance. Liposomes composed of saturated phospholipids with high Tm (e.g., DPPC, DSPC) form more rigid bilayers that reduce premature drug leakage and enhance circulatory stability, which is important for labile agents like zidovudine (AZT) [9]. In contrast, unsaturated lipids with low Tm impart greater fluidity, improving membrane fusion with target cells but reducing long-term stability [10]. PEGylation further modulates rigidity, generating a steric barrier that reduces opsonization while maintaining sufficient membrane flexibility for uptake by macrophage-rich lymphoid tissues key reservoirs of HIV persistence. These physicochemical attributes directly translate into improved pharmacokinetics. Liposomal zidovudine (AZT) has shown prolonged plasma retention and reduced bone marrow toxicity compared with the free drug [11]. When coated with polyethylene glycol (PEG), liposomes avoid rapid immune clearance and preferentially accumulate in macrophage-rich tissues such as the spleen and lymph nodes [12]. Animal studies confirm these advantages with PEGylated elastic liposomal zidovudine achieved 27-fold higher lymphoid drug levels in rats compared with free AZT [13, 14], while in non-human primates, a lipid-based nanoparticle combining lopinavir, ritonavir, and tenofovir reached > 50-fold higher intracellular concentrations in lymph-node mononuclear cells than conventional formulations [15]. Other vesicular carriers offer additional benefits. Niosomes, composed of non-ionic surfactants and cholesterol, are more stable and cost-effective than liposomes, while proniosomes with their dry precursors improve oral delivery. For example, efavirenz-loaded proniosomes (490 nm, 78% EE, − 19.8 mV) displayed biphasic release and significantly enhanced oral bioavailability in rats, with Cmax increasing nearly six-fold and AUC rising seven-fold compared with plain efavirenz [16]. Flexible systems such as transfersomes and ethosomes are designed to penetrate skin and mucosal barriers. Transfersomal zidovudine gels in rats produced a 2–3-fold increase in AUC compared with conventional gels. Ethosomes, enriched with ethanol for enhanced fluidity, have been applied in topical microbicides and transdermal delivery. A zidovudine ethosomal patch achieved > 70% in vitro release, > 78% ex vivo permeation, and a 189-fold higher bioavailability than the marketed product, without causing dermal irritation [17].

### Lipidic systems

Lipid-based enhance oral bioavailability, prolong systemic circulation, and enable sustained release by protecting ARVs from enzymatic degradation and first-pass metabolism. Solid Lipid Nanoparticles (SLNs) are composed of physiologically compatible solid lipids that remain crystalline at body temperature [18]. Their rigid lipid core provides controlled drug release, reduced drug leakage, and greater stability compared with vesicular systems. Lopinavir-loaded solid lipid nanoparticles (Lo-SLNs, 230 nm, − 27 mV) prepared by hot homogenization and ultrasonication achieved controlled release and markedly improved absorption. In rats, Lo-SLNs increased lymphatic drug transport 4.9-fold and oral bioavailability 2.1-fold over a conventional suspension. The formulation remained stable with a predicted shelf life of 21 months. Vaginal SLN gels have also been developed as pre-exposure prophylaxis (PrEP). Tenofovir-loaded SLNs incorporated into a hydrogel matrix enhanced mucosal penetration, prolonged drug residence, and provided more consistent protection in ex vivo tissue models [19]. Nanostructured Lipid Carriers (NLCs) are considered the “second generation” of lipid nanoparticles. Unlike SLNs, they combine solid and liquid lipids, creating an imperfect matrix that accommodates more drug and reduces the risk of drug expulsion during storage. Optimized formulations (10% drug loading, > 90% encapsulation efficiency) exhibited nanoscale size, good stability, and biphasic release with an initial burst (0–3 h) followed by sustained release over 24 h. Stability testing showed that both SLNs and NLCs remained physically stable for up to 8 weeks at 25 °C/60% RH, confirming their robustness as aqueous dispersions [20]. Lipid–Drug Conjugates represent a more advanced strategy, in which ARVs are covalently bound to lipid moieties, improving solubility, stability, and lymphatic transport. These conjugates bypass hepatic first-pass metabolism by entering systemic circulation via the lymphatic system, thereby enhancing oral bioavailability. Ritonavir-loaded solid lipid nanoparticles (RN-SLNs) optimized with soya lecithin as the lipid (highest solubility, 103.3 mg/g) and a Tween 80: Poloxamer 188 surfactant blend achieved a mean particle size of ~ 265 nm, encapsulation efficiency of 86%, and cumulative drug release of ~ 95%. The formulation exhibited biphasic release kinetics consistent with Higuchi’s model and improved oral bioavailability 4.3-fold compared with the marketed product, demonstrating its potential for enhanced ritonavir delivery [21].

### Polymeric systems

Polymeric nanocarriers are prepared from biodegradable or biocompatible polymers such as poly (lactic-co-glycolic acid) (PLGA), chitosan, or polyethyleneimine. Their versatility allows the encapsulation of small molecules, proteins, and nucleic acids, making them central to current HIV nanomedicine research [22]. Many ARVs, including efavirenz and tenofovir derivatives, possess carbonyl, amine, or hydroxyl groups capable of forming hydrogen bonds with PLGA’s ester linkages or chitosan’s amine and hydroxyl functionalities. These Strengthens drug–polymer association, increases loading efficiency, and slows early-phase release (“burst release”) [23]. PLGA degradation kinetics are central to sustained antiretroviral release and bioavailability in HIV nano-therapy. PLGA degrades through hydrolysis, and the rate is controlled by its lactic acid (LA) to glycolic acid (GA) ratio, molecular weight, and end-group chemistry. Higher GA content accelerates degradation due to increased hydrophilicity, while higher LA content slows breakdown and extends drug release. Likewise, low-molecular-weight and acid-terminated PLGA degrades faster, whereas high-molecular-weight and ester-terminated forms provide longer release profiles [24]. Additionally, during degradation, PLGA nanoparticles gradually develop an acidic micro-environment within their core. As water enters the matrix, it hydrolyses the polymer’s ester bonds, releasing lactic and glycolic acids. These acidic by-products accumulate faster than they can diffuse out, causing a gradual drop in the internal ph. The resulting acidification then amplifies the hydrolysis process an autocatalytic effect that accelerates polymer erosion from the inside outward [25].

In one study, efavirenz, lopinavir, and ritonavir were co-encapsulated into PLGA nanoparticles prepared by high-pressure homogenization. The optimized formulation achieved > 79% drug entrapment efficiency for each drug, with a mean size of ~ 138 nm and a surface charge of − 13.7 mV. The nanoparticles showed efficient uptake into both HeLa and H9 T cells, with sustained intracellular retention and minimal cytotoxicity over 28 days. In HIV-1–infected monocytic cells, treatment with cART nanoparticles led to significantly higher nuclear, cytoskeletal, and membrane drug concentrations compared to free drug solutions. Functionally, these nanoparticles strongly suppressed viral replication, with IC50 values for all three drugs below 31 nM [26]. These systems prolong circulation and reduce the risk of neurotoxicity by lowering the initial peak concentration (Cmax). Polymeric Micelles and Nanogels that target amphiphilic polymers to solubilize poorly water-soluble ARVs. Polymeric micelles enhance the dissolution and stability of drugs like efavirenz and lopinavir, while nanogels, formed from crosslinked hydrophilic polymers, can swell in biological fluids and release their payload in a controlled manner [27].

Furthermore, unlike passive carriers, some dendrimers, such as SPL7013 in VivaGel^®^, have intrinsic anti-HIV activity by electrostatically adhering to the viral envelope glycoprotein gp120, preventing the virus from activating CD4 receptors and infecting host cells [28]. This technique improves drug synergy and establishes dendrimers as dual-functional nano-therapeutics. Pharmacokinetically, dendrimer-drug conjugates improve retention duration and local bioavailability, particularly in mucosal applications [29]. However, their cationic surface charge, which promotes cellular uptake, has been linked to pro-inflammatory reactions and hemolysis. Surface modification methods, such as PEGylation or acetylation, are being used to balance bioactivity with biocompatibility to control these effects. Other examples of dendrimers in antiviral research include poly(amidoamine) (PAMAM) dendrimers, which have been under investigation for delivering nucleoside reverse transcriptase inhibitors (NRTIs) and siRNA, and polylysine dendrimers, investigated for gene delivery and antiviral applications [1].

### Nanocrystals

Nanocrystals are pure drug particles reduced to the nanoscale, usually ranging from 100 to 1000 nanometers, and stabilised with surfactants or polymers to prevent aggregation. Nanocrystals have been utilised in antiretroviral therapy to design long-acting injectable suspensions of drugs like rilpivirine and cabotegravir [30]. These formulations allow for extended drug release over weeks or months, lowering dose frequency and enhancing patient adherence. The FDA-approved long-acting injectable combination of cabotegravir and rilpivirine uses nanocrystal technology to keep therapeutic plasma levels stable with monthly or biweekly dosing [31, 32]. Nanocrystals also improve tissue penetration and dispersion, particularly in lymphoid tissues and macrophage reservoirs, which are important sites for HIV persistence. Nanocrystals, with their comparatively simple manufacturing process and scalability, are an appealing platform for enhancing the pharmacokinetics and therapeutic efficacy of antiretroviral drugs. Nanocrystal-based ARTs address a central challenge in HIV treatment, long-term adherence, which has remained a primary cause of treatment failure and resistance emergence. Nanocrystals also offer formulation advantages in pediatric ART, where taste masking, smaller dosing volumes, and lower excipient loads are essential for compliance [33]. However, their long-term stability can be compromised by Ostwald ripening, where smaller particles dissolve and redeposit onto larger ones, causing particle growth and altering release rates. On the other hand, polymorph conversion occurs when metastable crystalline forms transform to more stable but less soluble structures (34). Managing these two processes is essential to maintain predictable release kinetics and ensure the effectiveness of long-acting injectable ARTs.

### Inorganic systems

Metallic nanoparticles are tiny particles made of metals like gold, silver, and platinum. Magnetic nanoparticles are made of iron oxide (e.g., magnetite Fe₃O₄ or maghemite γ-Fe₂O₃) and have superparamagnetic properties [35]. Metallic nanoparticles, particularly gold (AuNPs) and iron oxide nanoparticles, are currently being investigated for multifunctional delivery systems that combine therapy with imaging (theranostics) or site-specific targeting [36]. In HIV research, gold nanoparticles have been conjugated with small interfering RNA (siRNA) or antiretroviral peptides to downregulate viral transcription in CNS-resident microglia, a reservoir for latent HIV [37]. These systems use both the enhanced cellular uptake properties of AuNPs and their potential to cross the blood-brain barrier (BBB) when surface-functionalized with targeting peptides. AuNPs coupled with siRNA targeting the HIV-1 Tat protein suppressed viral replication in microglial in vitro cells [38]. An important factor influencing the performance of metallic nanoparticles is protein corona formation. Upon exposure to biological fluids, proteins rapidly adsorb to the nanoparticle surface, creating a dynamic corona that can mask targeting ligands, alter surface charge, modify biodistribution, and increase recognition by the immune system [39]. This corona effectively defines the nanoparticle’s “biological identity,” often overriding its engineered properties. In HIV nanomedicine, uncontrolled corona formation can reduce targeting accuracy to tissues such as the CNS or lymphoid reservoirs and may accelerate clearance by macrophages. Surface modifications such as PEGylation are therefore used to minimize protein adsorption and improve circulation stability [40]. However, clinical translation of metallic and magnetic nanoparticles remains difficult due to concerns about long-term retention, biodistribution, and cytotoxicity, especially with repeated or high-dose administration. Surface modification with biocompatible polymers (e.g., polyethene glycol, PEG) is one strategy being investigated to improve safety profiles and evade immune recognition [6]. These nanoparticles can be administered through different routes, including oral, intravenous, intramuscular, and intranasal delivery, as summarised in Table 1. The routes of administration depend on the therapeutic goal and the nature of the target tissue.

**Table 1:** Classification of Nano-Formulations for HIV drug delivery

Classification	Examples	Routes of Administration	Description	Key Features	Examples / Applications in HIV	Advantages	Limitations / Challenges
Vesicular Systems	Liposomes, Niosomes, Transfersomes, Ethosomes, Proniosomes	Oral, IV, IM, topical, transdermal, mucosal	Spherical vesicles with phospholipid/surfactant bilayer enclosing aqueous core	Encapsulate both hydrophilic (core) & hydrophobic (bilayer) drugs; PEGylation improves stealth	Liposomal AZT; PEG-liposomes for macrophage targeting; Proniosomal Efavirenz; Ethosomal AZT patches	Prolonged circulation; reduced toxicity; improved lymphoid and mucosal delivery	Stability issues without PEG; costly manufacturing; refrigeration/shelf-life limits
Lipidic Systems	SLN, NLC, Nanoemulsions, Lipid Nanocapsules, LNPs (for RNA)	Oral, vaginal, topical, IM, SC	Solid/semi-solid lipids or emulsified droplets (no aqueous core)	High loading of lipophilic drugs; depot forming; protects drugs from enzymatic degradation	Lopinavir-SLN; Tenofovir-SLN PrEP gel; mRNA-LNP HIV vaccine platforms	Enhances oral bioavailability; long-acting depot; mucosal delivery suitability	Limited hydrophilic-drug loading; lipid polymorphism; processing complexity
Polymeric Systems	PLGA NPs, Polymeric micelles, Nanogels, Dendrimers (e.g., PAMAM, SPL7013)	Oral, IV, IM, mucosal, inhalation	Biodegradable/biofunctional polymer matrix systems	Sustained release; surface functionalization for targeting; carries small molecules & nucleic acids	PLGA-Efavirenz/Lopinavir/Ritonavir; SPL7013 (VivaGel^®^); PAMAM-siRNA carriers	Precise release control; targeted delivery; some dendrimers = intrinsic antiviral activity	Polymer toxicity; scale-up cost; cationic dendrimers cause irritation & inflammation
Nanocrystals	Pure drug nanocrystals (stabilized particles)	IM, SC	Pure drug particles sized 100–1000 nm stabilized with surfactants	Ultra-high drug loading; slow dissolution; long-acting injectable properties	Cabotegravir LA; Rilpivirine LA (FDA-approved)	Monthly/bi-monthly dosing; improved adherence; pediatric formulation flexibility	Injection-site reactions; limited to injectables; crystal stability must be controlled
Inorganic Systems	Gold NPs, Iron-oxide NPs, Silica NPs	IV, intranasal, topical	Metal or metal-oxide structures	Imaging + drug delivery (theranostics); surface functionalization for targeting	AuNP-siRNA to microglia; magnetically guided delivery; CNS-targeting conjugates	BBB penetration with ligands; real-time imaging; highly tunable surface chemistry	Long-term accumulation; cytotoxicity risk; challenging regulatory path
Hybrid / Composite Systems	Lipid–polymer hybrids, Polymer-drug conjugates, Core-shell systems	Oral, IV, IM	Combined organic + inorganic or lipid + polymer components	Combines stability of polymers with biomimicry of lipids; co-delivery capacity	Lipid-polymer hybrid ARV NPs; polymer–drug conjugates for sustained ARV release	Co-delivery of ARVs + LRAs/CRISPR; enhanced reservoir targeting; extended release	Complex fabrication; higher cost; regulatory ambiguity

### Hybrid/Composite nanoparticles

Hybrid or composite nanoparticles represent an advanced class of nanocarriers engineered by integrating two or more different material types combining organic components (such as lipids or polymers) with inorganic structures [41]. This hybridization approach make use of the complementary strengths of individual nanoparticle systems, resulting in formulations with enhanced physicochemical stability, improved biocompatibility, and superior drug-loading efficiency. hybrid nanoparticles offer refined control over drug release kinetics and biodistribution profiles by uniting the biomimetic properties of lipid systems with the structural stability. Hybrid nanoparticle platforms used in HIV therapy include lipid polymer hybrids, polymer–drug conjugates, metal–organic composites, and core shell nanostructures [42]. Lipid–polymer hybrid nanoparticles, for example, consist of a polymeric core surrounded by a lipid shell, facilitating sustained drug release from the core while the lipid layer enhances membrane fusion and cellular uptake. These systems have demonstrated significant potential for delivering hydrophobic antiretroviral agents, improving oral bioavailability, and enhancing accumulation in HIV reservoir sites such as lymphoid tissues and macrophages [4]. Polymer–drug conjugates, in contrast, covalently attach therapeutic molecules to biodegradable polymers, enabling precise drug activation and prolonged circulation time, thereby minimizing fluctuations in plasma drug concentrations that contribute to resistance. Hybrid nanocarriers offer unique advantages in multimodal HIV treatment strategies. Their structural versatility enables efficient co-delivery of multiple antiretroviral agents within a single platform, supporting simultaneous targeting of different viral replication pathways and reducing pill burden (41).

## Mechanisms of nanoparticle-mediated targeted drug delivery

Nanoparticle-based drug delivery systems (NDDS) have emerged as a leading tool in HIV treatment innovation. HIV treatments are administered via oral (tablets, capsules, syrups), injectable (intramuscular or subcutaneous long-acting nanoparticle formulations), and topical routes (gels or creams for prevention). Nanoparticle-based therapies primarily utilise oral and injectable routes, while topical applications are mainly for prophylaxis rather than treatment. Nanoparticles in HIV therapy mediate targeted drug delivery using the following mechanisms.

### Passive targeting

Passive targeting target enhanced permeability and retention (EPR) effect which allows nanoparticles to accumulate in tissues with leaky vasculature and impaired lymphatic drainage. These conditions are common in inflamed or infected sites where HIV reservoirs reside. A PEGylated elastic liposomal formulation of zidovudine (AZT) showed enhanced transdermal flux (119.5 vs. 99.8 µg/cm²/hr) and a 27-fold higher lymphoid tissue accumulation compared to free drug. Cellular uptake in MT-2 cells reached 88.9% vs. 27.1% for drug solution [43, 44]. Similarly, Lipid nanoparticles (LNPs) carrying indinavir (IDV) achieved higher lymph node drug levels, increased intracellular PBMC concentrations, prolonged plasma residence, restored CD4⁺ T cells, and suppressed viral RNA in primates. A subsequent triple-drug LNP (LPV/RTV/TFV) further improved lymphatic targeting, combining protease and reverse transcriptase inhibition to enhance potency and limit resistance [45, 46]. Comparable results were observed with solid lipid nanoparticles of lopinavir, where intestinal lymphatic transport studies revealed a 4.9-fold increase in lymphatic secretion and a 2.1-fold increase in systemic bioavailability compared to a drug suspension [47, 48]. Furthermore, transdermal transfersomal formulations of zidovudine produced two- to three-fold higher plasma drug exposure (AUC) compared with conventional gels, and ethosomal tenofovir gels improved mucosal retention in ex vivo vaginal tissues [49].

### Active targeting

Active targeting, in contrast, relies on surface modifications of nanoparticles with ligands such as antibodies, peptides, or sugars that bind selectively to receptors on target cells. This strategy ensures precise cellular uptake and enhanced drug accumulation within reservoir cells [50]. For example, mannose-functionalized nanoparticles have been shown to achieve two- to four-fold higher uptake by macrophages and dendritic cells compared to unmodified particles [51]. This demonstrates their ability to concentrate antiretrovirals in monocyte/macrophage reservoirs where HIV often persists. Similarly, nanoengineered CD4⁺ T cell membrane-coated nanoparticles (TNPs) demonstrated broad neutralizing activity against 125 HIV-1 strains, with a mean IC₈₀ of 819 µg/mL. Beyond neutralizing cell-free virus, TNPs selectively bound HIV-infected CD4⁺ T cells and macrophages, inducing autophagy that inhibited viral release and reduced cell-associated HIV-1 [52]. Active targeting has also been extended to CNS delivery, where transferrin- or ApoE-modified nanoparticles improved brain penetration, producing two- to three-fold higher brain-to-plasma ratios compared with unmodified carriers in rodent models [53]. A key determinant of reproducible receptor-mediated uptake is ligand surface density, typically measured as ligands per nm². Studies show that effective targeting usually occurs within a narrow range approximately 0.01–0.1 ligands/nm² which provides sufficient receptor engagement without causing steric hindrance or rapid clearance [54]. Densities below this range reduce binding efficiency, while higher densities disrupt nanoparticle mobility and can trigger immune recognition. Precise control of ligand density is essential for ensuring consistent biodistribution and predictable therapeutic performance in clinically translatable active-targeting systems.

### Exosomal escape and Exosome-Mimicking delivery

Exosomes are natural vesicles that mediate intercellular communication and internalized by CD4⁺ T cells, macrophages, and dendritic cells. Nanoparticles engineered to mimic exosomes, or functionalized with exosomal surface proteins, target these pathways to bypass lysosomal degradation and achieve efficient intracellular drug release. Beyond endosomal escape, nanoparticles can also target or mimic the exosomal pathway to enhance drug delivery to HIV reservoir cells. Exosomes are naturally secreted extracellular vesicles that carry proteins, nucleic acids, and lipids between cells, playing a central role in intercellular communication [55]. Nanoparticles designed with exosome-like lipid membranes or decorated with exosomal surface proteins can be preferentially internalized by HIV target cells such as CD4⁺ T cells and macrophages. This mechanism allows them to evade lysosomal degradation, extend circulation time, and achieve precise intracellular delivery. For example, exosome-mimicking nanoparticles loaded with antiretrovirals showed 2–3 fold higher uptake in macrophages compared to conventional liposomes, while reducing drug loss through lysosomal degradation [56]. Similarly, exosome-derived vesicles carrying siRNA against HIV Tat and Rev genes achieved over 70% knockdown of viral RNA in latently infected cell lines, outperforming synthetic lipid nanoparticles [57]. Exosomal escape mechanisms have also been harnessed to deliver CRISPR-Cas9 complexes into resting CD4⁺ T cells, enabling proviral DNA excision with higher efficiency and reduced cytotoxicity compared to standard polymeric carriers. A key limitation of exosome-mimicking nanoparticles is batch heterogeneity, especially variability in lipid composition and surface protein levels. Small differences in the abundance or orientation of exosomal proteins (e.g., CD9, CD63, CD81) can change uptake efficiency and targeting accuracy, making reproducibility difficult. This variability remains a major barrier to standardization and clinical translation [58].

### Shock and kill strategy

The “shock and kill” approach aims to eliminate latent HIV reservoirs by first reactivating (“shocking”) the silent provirus using latency-reversing agents (LRAs), followed by killing the reactivated cells with antiretroviral drugs or immune clearance. Nanoparticles play a critical role by co-delivering LRAs and ARVs to the same reservoir sites, ensuring synchronized action [59]. For example, polymeric nanoparticles encapsulating romidepsin (LRA) with tenofovir have shown effective reactivation of latent virus in vitro, while simultaneously blocking new rounds of infection [60]. An mRNA–lipid nanoparticle (LNP X) was engineered to deliver mRNA into hard-to-transfect resting CD4⁺ T cells without inducing toxicity or activation. When encapsulating mRNA encoding HIV Tat, LNP X significantly enhanced viral transcription in ex vivo CD4⁺ T cells from people living with HIV [61]. The same platform also enabled delivery of CRISPR activation machinery to modulate viral and host genes, demonstrating its potential for nucleic acid-based “shock and kill” strategies targeting latent HIV reservoirs.

### Block and lock strategy

In contrast, the “block and lock” strategy aims to permanently silence HIV proviral DNA to achieve functional cure without reactivation. Nanoparticles facilitate this by delivering gene-editing tools (CRISPR-Cas9, siRNAs, or shRNAs) and transcriptional repressors directly into HIV-infected cells [62]. For example, lipid nanoparticles carrying CRISPR-Cas9 targeting HIV long terminal repeat (LTR) regions successfully excised proviral DNA in animal models, reducing viral rebound after treatment cessation [63]. Similarly, dendrimer-based carriers have been used to deliver siRNAs that downregulate Tat and Rev proteins, key transcriptional activators of HIV, effectively locking the virus in a dormant state [64, 65]. Alongside CRISPR-based repression, Tat/TAR decoy oligonucleotide nano-delivery represents another important “block and lock” strategy. In this approach, nanoparticles deliver synthetic oligonucleotides that mimic the TAR region of HIV RNA, allowing them to bind and capture the Tat protein. Hence, Tat is required to initiate HIV transcription, blocking its interaction with TAR helps maintain the virus in a deeply silent state. Nanoparticle delivery improves the stability and uptake of these decoys, supporting long-term viral suppression without activating T cells [66].

## Release pattern of nanoparticles mediated drug delivery in HIV treatment

The therapeutic success of nanoparticle-mediated drug delivery in HIV treatment depends on how effectively the carrier’s control and direct the release of antiretroviral drugs (ARVs).

### Controlled and sustained release

This approach involved the encapsulated ARVs within a nanoparticle matrix where they are released gradually over days, weeks, or months. This strategy maintains plasma concentrations above the therapeutic threshold for prolonged durations reducing the need for frequent dosing. For example, long-acting nanosuspensions of cabotegravir and rilpivirine, which are now approved for clinical use [67]. Administered intramuscularly, these formulations sustain plasma concentrations for up to 8 weeks, effectively transforming HIV management from daily oral pills to bi-monthly injections [68]. Preclinical studies with PLGA-based nanoparticles carrying tenofovir or efavirenz have demonstrated detectable plasma levels in rodent with release duration influenced by polymer composition (e.g., lactide: glycolide ratio) and particle size [69]. The clinical advantage of sustained release is enhanced adherence, a challenge in HIV therapy where missed doses result in viral rebound and resistance. NDDS directly address this barrier By reducing dosing frequency. However, sustained release systems introduce new challenges with once administered, depot formulations cannot be easily reversed in cases of hypersensitivity or adverse drug reactions. Furthermore, injection-site reactions, pain, and local inflammation have been reported in trials limiting their long-term acceptability [70].

### Stimuli-Responsive release

Stimuli-responsive nanoparticles represent an emerging frontier in HIV drug delivery, designed to release drugs in response to specific biological triggers. These stimuli include pH, enzymatic activity, and redox potential, which vary between normal and diseased tissues [71]. pH-responsive systems destabilize at pH 5.0–6.0, the acidity found in early and late endosomes of macrophages, enabling selective payload release in one of HIV’s major reservoirs. pH-sensitive liposomes destabilize under acidic conditions, found in endosomal compartments of macrophages one of the principal reservoirs for HIV. They enhance drug delivery specifically to infected cells by selectively releasing their payload in these compartments. Similarly, redox-responsive micelles are triggered by the elevated intracellular glutathione (GSH) levels of activated immune cells 2–10 mM cytosolic GSH versus 2–10 µM extracellular GSH which cleave disulfide linkers and induce rapid carrier disassembly [72, 73]. Enzyme-responsive nanoparticles often rely on lysosomal hydrolases such as cathepsin B, which reach active concentrations of 50–200 nM inside macrophage lysosomes, sufficient to degrade peptide- or polymer-based linkers and release ARVs intracellularly [74]. The advantage of stimuli-responsive systems is their potential to minimize systemic exposure while maximizing delivery to sites of viral persistence. Furthermore, external stimuli such as light and temperature are being explored to control nanoparticle drug release in HIV therapy. Light-responsive nanocarriers release their payload when exposed to specific wavelengths, enabling highly localized activation and minimizing systemic exposure. Thermosensitive nanoparticles similarly respond to mild temperature changes, allowing controlled release at sites of inflammation or through externally applied heat. Although still in early investigation for HIV compared to cancer research, these external trigger systems offer precise, on-demand drug delivery and represent a promising complementary strategy for targeted antiretroviral release [75].

### Targeted release in viral reservoirs

One of the defining challenges in HIV cure research is the persistence of viral reservoirs in macrophages, lymph nodes, and the central nervous system (CNS). Conventional oral ART achieves effective plasma suppression but often fails to eliminate the virus from these sanctuaries. Nanoparticles enhance drug delivery to reservoirs through passive targeting (size and biodistribution) and active targeting (ligand functionalization) [76]. For example, mannose-decorated polymeric nanoparticles target macrophage mannose receptors to enhance uptake, while transferrin-functionalized liposomes have shown improved transport across the blood–brain barrier [77]. In macaque studies, nanoformulated atazanavir achieved significantly higher concentrations in lymph nodes than conventional drug formulations, highlighting the reservoir-targeting potential [78]. Similarly, solid lipid nanoparticles have been reported to increase efavirenz penetration into brain tissue, which is particularly important for controlling HIV-associated neurocognitive disorders [79]. In addition to in vivo studies, advanced in vitro platforms are increasingly used to evaluate nanoparticle penetration into CNS reservoirs. Brain organoid systems and microfluidic blood–brain barrier (BBB-on-a-chip) models have enabled more physiologically relevant assessment of nanoparticle transport, neural uptake, and potential neurotoxicity [80]. These models better mimic human BBB structure and neural microenvironment than traditional cell monolayers, providing valuable insight into nanoparticle behaviour before animal or clinical testing. Their use has strengthened preclinical evaluation pipelines for CNS-directed HIV nano-therapies and supports rational design of carriers capable of targeting brain reservoirs and mitigating HIV-associated neurocognitive complications [81]. A continuing limitation in CNS-directed HIV nano-therapy is the activity of efflux transporters, particularly P-glycoprotein (P-gp) and Breast Cancer Resistance Protein (BCRP). Even when nanoparticles successfully cross the BBB, these pumps can actively expel antiretroviral drugs back into the bloodstream, reducing net CNS accumulation. Many ARVs including efavirenz, lopinavir, and dolutegravir are substrates of P-gp or BCRP, which restricts their therapeutic levels in brain reservoirs [82].

## Designing and formulation of nanoparticles for HIV drug delivery

**Fig. 3:**
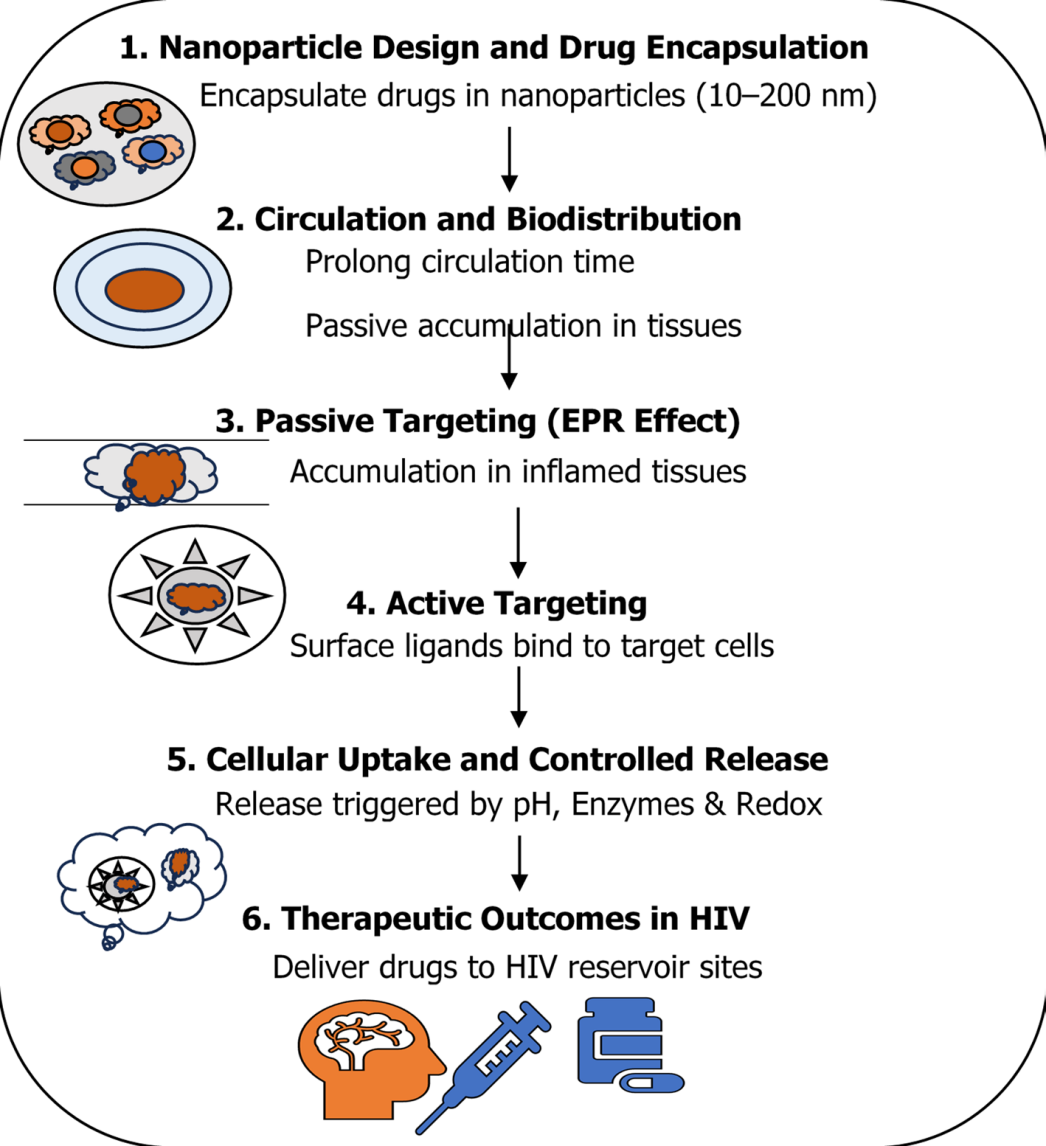
Formulation and Design of Nanoparticles for HIV Drug Delivery. Nanoparticles encapsulate antiretroviral drugs (10–200 nm) to enhance solubility and stability (Step 1). Following administration, they prolong circulation time and improve biodistribution with passive tissue accumulation (Step 2). Through the enhanced permeability and retention (EPR) effect, they preferentially accumulate in inflamed or reservoir tissues (Step 3). Active targeting further refines delivery by using surface ligands to bind HIV-infected cells (Step 4). Once internalized, nanoparticles enable controlled drug release triggered by pH, enzymatic activity, or redox conditions (Step 5). Collectively, these processes improve therapeutic outcomes by delivering drugs to difficult-to-reach HIV reservoir sites, enhancing efficacy and adherence (Step 6)

Designing and formulating nanoparticles tailored for HIV drug delivery involves careful consideration of physicochemical properties, surface modifications, and drug loading techniques to optimise therapeutic efficacy. This approach aims to improve drug pharmacokinetics, increase accumulation in viral reservoirs, and ultimately contribute to more effective viral suppression and potential elimination. Figure 3 summarises the approach to design and formulation of Nanoparticles for HIV drug delivery.

### Material selection

Lipid-based carriers such as liposomes and solid lipid nanoparticles (SLNs) have been employed to encapsulate hydrophobic ARVs like efavirenz and lopinavir. For example, Solid lipid nanoparticles (SLNs) have also been applied to enhance the oral delivery of ritonavir. In rats, optimized ritonavir-loaded SLNs prepared by hot homogenization followed by ultrasonication achieved a particle size of ~ 265 nm, an entrapment efficiency of 86%, and biphasic drug release following Higuchi’s model. In vivo pharmacokinetic evaluation showed that these SLNs increased oral bioavailability by 4.3-fold compared with the marketed formulation, highlighting their ability to overcome poor solubility and first-pass metabolism [83]. Liposomes, when PEGylated prolong circulation and facilitate preferential uptake in macrophage-rich tissues [84]. These findings support the potential of lipid-based systems to enhance ARV delivery into viral reservoirs. However, results remain species-specific, route-dependent, and formulation-dependent. Polymeric nanoparticles such as poly (lactic-co-glycolic acid) (PLGA) and chitosan offer better degradation profile and enable sustained release. PLGA nanoparticles encapsulating dolutegravir have been reported to achieve detectable plasma levels in animal models [85]. Chitosan nanoparticles further provide mucoadhesive properties that enhance gastrointestinal absorption of encapsulated drugs, making them attractive for oral formulations. Inorganic carriers such as gold or silica nanoparticles are less common for systemic HIV therapy but have been investigated as theragnostic platforms. Their large surface-area-to-volume ratio facilitates multivalent drug or ligand loading, and their intrinsic optical properties allow concurrent imaging [86].

### Nanoparticle synthesis techniques for HIV drug delivery

Several well-established and emerging techniques have been used to facilitate the production of nanoparticles with desirable efficiency. Among these techniques, nanoprecipitation is widely used for hydrophobic antiretrovirals (ARVs) such as saquinavir [87]. This technique involves dissolving the drug and polymer in a water-miscible organic solvent. This is followed by introducing the solution into an aqueous phase, where nanoparticles form spontaneously by rapid solvent exchange and precipitation. Another technique used in nano-synthesis is the Emulsification Solvent Evaporation [88]. It provides greater flexibility by accommodating both hydrophilic and hydrophobic drugs. The drug-polymer mixture is emulsified into an aqueous phase, followed by solvent evaporation to yield nanoparticles. This method is commonly used for preparing poly (lactic-co-glycolic acid) (PLGA)-based formulations. Ionic Gelation is another technique that offers a mild and efficient route for synthesising chitosan-based nanoparticles. The technique is suitable for nucleic acid therapeutics like siRNA and mRNA. It relies on electrostatic interactions between the positively charged chitosan and negatively charged crosslinkers such as tripolyphosphate (TPP). This facilitates nanoparticle formation without harsh solvents or elevated temperatures [85]. High-pressure homogenization and Ultrasonication are mechanical techniques used to produce solid lipid nanoparticles and nano-emulsions. These techniques apply shear forces to reduce particle size while preserving drug encapsulation efficiency. This feature makes them specifically suitable for thermolabile ARVs or lipid-based payloads [89]. Microfluidics-based synthesis technique allows precise control over nanoparticle size, surface characteristics, and polydispersity. It is particularly suitable for synthesising lipid nanoparticles used in RNA delivery systems and CRISPR-Cas9 gene-editing platforms due to its size reduction ability [90].

### Surface modification and functionalization for Cell-Specific targeting

Surface modification of nanoparticles is important in achieving cell-specific targeting in HIV therapy. Nanoparticles are functionalized with a biological ligand such as mannose, transferrin, or anti-CD4 antibodies. This process enables receptor-mediated uptake by HIV reservoir cells, including macrophages, brain endothelial cells, and CD4⁺ T lymphocytes [91]. The modifications enhance intracellular drug delivery while minimising systemic toxicity. For example, mannose-functionalized nanoparticles exploit the overexpression of mannose receptors on macrophages, leading to enhanced uptake, improved drug retention, and efficient suppression of viral replication in lymphoid and CNS tissues [92, 93]. Similarly, aptamer-conjugated nanoparticles provide highly specific delivery of antiretroviral drugs (ARVs) to CD4⁺ T cells, which are reservoirs for latent HIV. This strategy ensures targeted drug accumulation in lymphoid tissues while reducing off-target effects. In addition to ligand conjugation, polyethene glycol (PEG) is frequently used to pegylate nanoparticles, prolonging systemic circulation by reducing immune recognition and clearance [93]. These combined surface-coated strategies have been reported to improve pharmacokinetics, biodistribution, and therapeutic efficacy of ARV-loaded nanoparticles, as summarised in Table 2.

**Table 2:** Examples of functionalised nanoparticles for HIV drug delivery

Nanoparticles	Materials	Drug delivery	Functionalization		Target	Features	
Liposomes	Phospholipids	Tenofovir, Lopinavir	PEGylation	CD4 + T cells, Lymphoid tissues			prolonged circulation, high drug encapsulation efficiency
Polymeric Nanoparticles	PLGA (Poly-lactic-co-glycolic acid)	Dolutegravir, Zidovudine	Mannose, Folic acid	Macrophages, HIV reservoirs			Biodegradable, sustained release
Solid Lipid Nanoparticles	Solid lipid	Efavirenz, Ritonavir	PEGylation	HIV-infected cells			Stable at body temperature, enhanced bioavailability
Micelles	Amphiphilic polymer	Lopinavir	Mannose	Macrophages			High drug-loading capacity for hydrophobic drugs
old Nanoparticles (AuNPs)	Gold	Antiretroviral peptides	CD4 aptamers, CCR5 antibodies	CD4 + T cells			High specificity, potential for diagnostic and therapeutic dual roles
Dendrimers	PAMAM (Polyamidoamine)	Zidovudine, Saquinavir	Surface modification with mannose	Macrophages, Lymphoid tissues			Highly branched structure, capable of encapsulating or binding multiple drugs
Chitosan Nanoparticles	Chitosan	Zidovudine, Tenofovir	Mannose	Macrophages			Biodegradable, pH-sensitive, suitable for oral and intravenous delivery
Carbon Nanotubes (CNTs)	Carbon-based	Efavirenz, Lamivudine	PEGylation, Peptide conjugation	HIV reservoirs			High drug-loading capacity, potential for crossing cellular membranes

Furthermore, these functionalization strategies are directly linked to the challenge of HIV latency. Latent reservoirs persist in sanctuary sites such as the CNS, GALT, and secondary lymphoid organs, which remain poorly accessible to conventional ART. Functionalized nanoparticles such as transferrin-coated particles to cross the blood–brain barrier or mannose-modified carriers for macrophage targeting in GALT enable enhanced drug transport into these protected compartments [91]. These systems improve viral suppression within reservoirs and reduce the risk of rebound after treatment interruption by concentrating ARVs in long-lived reservoir cells. Thus, surface modification improves biodistribution and represents an important strategy for overcoming the central barrier of latency in HIV treatment. PEGylation is widely used to prolong circulation, it can also trigger the formation of anti-PEG antibodies, particularly after repeated dosing. These antibodies increase nanoparticle clearance through the accelerated blood clearance (ABC) phenomenon, reducing systemic half-life, altering biodistribution, and diminishing targeting efficiency [94]. This effect has been reported across PEGylated liposomes, polymeric nanoparticles, and protein–drug conjugates. As a result, newer strategies such as zwitterionic coatings or alternative hydrophilic polymers are being explored to retain stealth properties without inducing anti-PEG immune responses [95].

### Physicochemical optimisation: Size, Charge, and morphology

The physicochemical properties of nanoparticles, particularly size, surface charge, and morphology, play an important role in their biological behaviour. These include biodistribution, cellular uptake, and clearance, which are essential considerations for effective HIV drug delivery. Nanoparticles within the range of 50–150 nm in size are optimal for avoiding rapid renal clearance and leveraging the enhanced permeability and retention (EPR) effect [96]. This size range facilitates passive targeting by allowing nanoparticles to accumulate in lymphoid tissues and inflamed vasculature. Cationic (positively charged) nanoparticles promote enhanced cellular uptake due to electrostatic interactions with negatively charged cell membranes. However, this increased uptake can be accompanied by higher cytotoxicity and faster clearance by the immune system. Surface neutralisation strategies, such as polyethene glycol (PEG) coating (PEGylation), are used to control these effects. PEGylation reduces opsonisation by serum proteins, prolonging systemic circulation and improving nanoparticle stability [97]. Morphologically, Spherical nanoparticles are generally preferred for their ease of large-scale manufacturing and predictable biodistribution profiles. Nonetheless, alternative shapes such as rod-shaped or worm-like nanoparticles have demonstrated improved margination and retention in mucosal tissues [98]. In addition to size and morphology, surface charge (ζ-potential) strongly influences nanoparticle biodistribution, particularly lymphatic transport and immune recognition. Near-neutral or mildly negative ζ-potential favours lymphatic uptake and reduces opsonization, promoting accumulation in lymphoid tissues where HIV reservoirs persist. Conversely, highly cationic particles enhance cellular binding but increase immune activation and clearance, highlighting the importance of ζ-potential optimization to balance immune evasion and targeted delivery in HIV nano-therapy [99].

## Safety of nanoparticle-based antiretroviral systems

Safety remains the one the major determinant of whether nanoparticle-based antiretroviral drug delivery systems (NDDS) can progress from experimental promise to clinical reality. Their efficacy and pharmacokinetic advantages have been repeatedly demonstrated, clinical translation is limited by uncertainties around cytotoxicity, immunogenicity, and long-term accumulation.

### Cytotoxicity

Cytotoxicity is of nanoparticle depend on the physicochemical properties of nanoparticles, including size, surface charge, and composition. Biodegradable carriers such as PLGA and chitosan are generally metabolized into lactic and glycolic acid or excreted without causing overt toxicity at therapeutic doses. For example, mouse models demonstrate tolerability of PLGA nanoparticles up to 100 mg/kg, with minimal hepatic or renal damage when polydispersity is controlled [100]. By contrast, cationic dendrimers and metallic nanoparticles demonstrate significantly lower safety margins. Cationic PAMAM dendrimers exhibit LD50 values between 100 and 200 mg/kg in rodents, with hepatotoxicity, nephrotoxicity, and oxidative stress commonly observed [101]. Similarly, gold nanoparticles exceeding 50 mg/kg in mice have been linked to elevated alanine transaminase (ALT) levels and histological evidence of hepatic damage [102]. In humans, liposomal formulations such as pegylated liposomal doxorubicin demonstrate reduced systemic cytotoxicity compared to free drug [103]. However, infusion-related adverse events have been reported, highlighting that nanoparticle encapsulation alters but does not eliminate toxicity [104].

### Immunogenicity

Nanoparticles interact with the immune system in unpredictable ways. Complement Activation–Related Pseudoallergy (CARPA) is a well-characterized reaction triggered by liposomes, micelles, and iron oxide nanoparticles. Porcine models have reproduced cardiopulmonary distress following nanoparticle infusion, which correlates with human hypersensitivity syndromes [105]. Clinically, pegylated liposomal doxorubicin has been associated with infusion reactions in approximately 7% of patients, while ferumoxytol carries warnings for hypersensitivity-related adverse events [106]. An additional concern is the increase in the prevalence of anti-PEG antibodies (APAs), estimated at 25–44% in human cohorts [107]. These antibodies increased nanoparticle clearance (the “ABC phenomenon”) and increase the risk of hypersensitivity reactions upon repeated dosing [108].

### Long-Term accumulation and clearance

Clearance pathways vary by nanoparticle composition with biodegradable polymers such as PLGA undergo hydrolysis, with metabolites entering the Krebs cycle and being eliminated as carbon dioxide and water [109]. In contrast, metallic nanoparticles and certain synthetic polymers persist within the reticuloendothelial system (RES), accumulating in the liver, spleen, and lymph nodes [110]. A biodistribution in mice studies reveal persistent organ deposition weeks after administration of silver and iron oxide nanoparticles, raising concern about subclinical organ burden [111]. Additionally, nanoparticles that are largely biodegradable may exhibit altered clearance when repeatedly administered. Liposomal carriers remain encapsulated in plasma for extended periods (half-life of pegylated liposomal doxorubicin ≈ 55 h, with > 90% drug encapsulated during circulation), leading to prolonged RES exposure [112].

## Manufacturing, regulatory, and cost-effectiveness limitations

Manufacturing scalability, regulatory uncertainty, and cost-effectiveness considerations form a structural barriers that determine whether NDDS can achieve widespread adoption.

### GMP manufacturing challenges

The production of NDDS requires precision methods such as microfluidics, solvent evaporation, and ligand conjugation, each demanding specialized infrastructure and technical expertise. Unlike conventional oral formulations, where quality control is straightforward, nanoparticles must be characterized by dynamic light scattering, zeta potential analysis, and cryo-electron microscopy to ensure consistency [113]. Batch-to-batch variability in particle size or surface charge can alter pharmacokinetics and toxicity, affect both safety and efficacy. Compliance with Good Manufacturing Practices (GMP) is therefore resource-intensive, and the lack of standardized international guidelines complicates large-scale production [114]. To address these constraints, scalable fabrication approaches are being developed. Continuous-flow microfluidic systems enable controlled nanoparticle assembly with improved batch uniformity and reduced solvent consumption, making them suitable for large-scale manufacturing compared to traditional batch methods [115]. Similarly, spray-drying of nanosuspensions allows conversion of liquid nanoparticle formulations into dry, stable powders for easier storage, transport, and reconstitution, supporting industrial-scale production [116]. These technologies represent promising strategies to overcome current GMP limitations and facilitate commercial translation of nanoparticle-based HIV therapies.

### Regulatory barriers

Regulatory agencies worldwide continue to adapt legacy frameworks initially developed for small molecules and biologics to the complexities of nanomedicines. Existing approval standards do not fully capture nanoparticle-specific pharmacokinetics, biodistribution, immunogenicity, and long-term safety, creating uncertainty and prolonging evaluation timelines [117]. The U.S. FDA has issued several nanotechnology guidance documents emphasizing material characterization, critical quality attributes, and risk-based assessment, while the EMA provides more structured guidance for nanocarriers and liposomal products, including scientific advice for nanoparticle excipients and surface modifications. Despite these advances, harmonization remains limited [118]. Recent ICH Q13 guidelines on continuous manufacturing now offer an opportunity to streamline nanoparticle production by supporting real-time process monitoring and consistent product quality, yet integration into nanomedicine regulatory pathways is still evolving [119]. These gaps create challenges for complex multi-drug nano-formulations used in HIV therapy, which require additional scrutiny for stability, drug–drug interactions, and clinical endpoints.

### Economic and logistical barriers

Even where NDDS demonstrate clinical benefit, their economic feasibility present another challenge. Compared to conventional oral antiretroviral therapy, NDDS cost 2–10 times more per dose, a disparity driven by expensive raw materials, specialized equipment, and quality control requirements [118]. For long-acting formulations requiring cold chain storage, the logistical challenges in low- and middle-income countries (LMICs) are significant, adding further costs. This creates a risk of widening inequities in HIV treatment access, particularly where funding and procurement systems are already strained. Cost-effectiveness analyses are urgently needed to weigh the long-term benefits of improved adherence, reduced hospitalizations, and decreased viral transmission against the short-term costs of NDDS. Without targeted reforms such as tiered pricing, technology transfer, pooled procurement, or licensing agreements, access in resource-limited settings will remain limited [120].

## Nanoparticles in HIV diagnostics and Image-Guided therapy

Beyond drug delivery, nanoparticles can play important diagnostics and image-guided therapy, expanding their role in HIV management from treatment to detection and monitoring. Conventional HIV diagnostic platforms rely on ELISA, PCR, and rapid immunoassays which are sensitive. Nanoparticles provide an innovative alternative through their unique optical, magnetic, and electrochemical properties for point-of-care diagnostics. For example, gold nanoparticles (AuNPs) conjugated with HIV antibodies or nucleic acid probes have been used in colorimetric assays, enabling naked-eye detection of viral proteins or RNA at femtomolar concentrations. These low-cost and portable assays are particularly relevant for early diagnosis and community-based screening in low- and middle-income countries (LMICs). Similarly, quantum dots (QDs) and magnetic nanoparticles (MNPs) have been employed in biosensor platforms for multiplexed detection of HIV biomarkers, offering higher sensitivity and faster turnaround compared to standard assays. Additionally, nanoparticles enable image-guided therapy (theranostics), combining diagnostic imaging with therapeutic delivery in a single platform. Iron oxide nanoparticles can simultaneously deliver antiretroviral drugs and provide magnetic resonance imaging (MRI) contrast. This allows clinicians to monitor biodistribution and reservoir targeting in real time. This dual functionality enhances treatment precision and provides valuable pharmacodynamic understanding that conventional ART cannot offer. However, technical barriers include standardization of nanoparticle-based assays, stability of probes under field conditions, and regulatory acceptance of nano-diagnostics. From a therapeutic perspective, the high cost and infrastructural requirements of imaging platforms (e.g., MRI, PET) restrict their applicability in LMICs, where HIV burden is highest.

## **Clinical applications and translational evidence**

The transition of nanoparticle-based drug delivery systems (NDDS) from laboratory research into clinical practice has transformed HIV prevention and therapeutic strategies, as outlined in Table 3. These systems have been shown to increase patient adherence, extend drug half-life, reduce dose frequency, and optimise outcomes. Nanomedicine has been supported by regulatory approvals, pivotal Phase III clinical trials, and real-world implementation data. NDDS has moved beyond preclinical promise into mainstream clinical utility. One of the most notable clinical breakthroughs is the development and global rollout of long-acting injectable antiretroviral therapy (LA-ART) [121]. The approved combination of cabotegravir and rilpivirine (Cabenuva^®^) represents a major advance. They are formulated as nanosuspensions with slow-release properties, allowing monthly or bimonthly dosing. Results from the ATLAS and FLAIR trials demonstrated non-inferiority to daily oral ART, with over 90% of participants maintaining virologic suppression at 48 and 96 weeks [122]. Patients also reported improved convenience, satisfaction, and adherence factors essential for successful long-term disease management. These findings have prompted updates in WHO guidelines and informed the integration of LA-ART into national HIV programs. The durability and simplified dosing of LA-ART align with public health goals to reduce clinic burden and decentralise care delivery in resource-limited regions. Long-acting formulations for pre-exposure prophylaxis (PrEP), such as injectable cabotegravir (Apretude™), have shown superior efficacy compared to daily oral tenofovir/emtricitabine (TDF/FTC). In the HPTN 083 and 084 trials, injectable PrEP reduced HIV acquisition by 66–89% depending on population and adherence level [123]. These advancements are particularly suitable for high-risk groups such as young women, sex workers, and men who have sex with men, who often face barriers to daily drug adherence. Furthermore, nanoparticle-enhanced intravaginal rings and nanogels incorporating dapivirine or tenofovir have demonstrated promising mucosal retention. Moreover, Nano-formulated protease inhibitors like lopinavir/ritonavir in solid lipid nanoparticle suspensions offer improved taste, enhanced bioavailability, and reduced dosing frequency [124]. These pediatric-friendly formulations could be seamlessly integrated into school-based or community ART programs, lowering drug burden and improving caregiver and patient satisfaction. Furthermore, early-phase trials are evaluating nanoparticles co-loaded with latency-reversing agents (e.g., romidepsin) and antiretrovirals to target and reactivate latent virus while simultaneously neutralizing released virions. These systems utilise polymeric nanoparticles and dendrimer conjugates to penetrate deep anatomical reservoirs [125]. Currently, nanoparticle-based ointments for HIV treatment are not widely established or clinically approved. However, research is ongoing in this area, especially for topical microbicides aimed at preventing sexual transmission of HIV. To further illustrate this translational progress, Table 4 presents a comprehensive overview of recent and real-world case studies across different nanoparticle classes, highlighting their practical relevance and emerging clinical applications.

**Table 3:** Clinical applications of Nanoparticle-Based antiretroviral delivery systems

Formulation / Platform	Drug(s)	Trial / Study	Population	Key Findings	Clinical Impact	Reference
Long-acting injectable nanosuspensions (LA-ART)	Cabotegravir + Rilpivirine (Cabenuva^®^)	ATLAS & FLAIR (Phase III)	HIV-positive adults	> 90% virologic suppression at 48–96 weeks; non-inferior to daily oral ART	Monthly/bimonthly dosing; WHO guideline updates	[147]
Long-acting injectable PrEP	Cabotegravir (Apretude™)	HPTN 083 & 084	High-risk populations (MSM, women)	66–89% lower HIV incidence vs. daily oral TDF/FTC	First injectable PrEP approved; improved adherence	[148]
Intravaginal rings & nanogels	Dapivirine, Tenofovir	IPM & MTN trials	Women at high risk	Prolonged mucosal drug retention; improved local protection	Promising for female-controlled prevention	[149]
Pediatric nanoformulations	Lopinavir/Ritonavir SLN suspensions	Early-phase clinical evaluation	HIV-positive children	Improved taste, higher bioavailability, reduced dosing frequency	Better adherence, caregiver acceptability	[150]
Nanoparticles with LRAs + ARVs	Romidepsin + ARV combos	Ongoing early-phase	Adults with latent HIV	Reactivates latent virus + neutralizes released virions	Potential ‘shock and kill’ cure strategy (translational)	[151]

**Table 4:** Comprehensive Case-Study table: Nano-Formulations for HIV therapy

Nanocarrier Class	Case Study & Year	Formulation & Strategy	Mechanism/Target	Key Outcome / Findings	Reference
Vesicular Systems (Liposomes, Niosomes, Ethosomes, Transfersomes)	PEG-Ethosomal Zidovudine Patch	PEG-ethosomal transdermal patch for AZT	Transdermal/lymphatic targeting	189-fold increase bioavailability, > 78% permeation, non-irritant	[152]
	Tri-ARV Liposomal Nano-formulation in Primates (2024)	Liposomes delivering lopinavir/ritonavir/tenofovir	Lymph node reservoir delivery	> 50-fold increase lymph-node drug levels in primates	[153]
Lipid-Based Nanocarriers (SLN, NLC)	Efavirenz SLN/NLC (2019)	SLN/NLC formulation for EFV	Sustained delivery & stability	Increase stability, improved drug release & tissue targeting	[154]
	Ritonavir SLN Oral System (2025)	SLN-based ritonavir oral nanosystem	Oral lymphatic uptake	4-fold enhance oral bioavailability	[155]
Polymeric Nanoparticles (PLGA, Polymer micelles)	Tenofovir PLGA-NPs (2017)	PLGA nanoparticles for oral TDF	Mucosal & intracellular delivery	6-fold increase absorption & mucosal uptake	[156]
Polymeric Nanoparticles (PLGA, vitro, ex vivo and in vivo evaluation)	Combo-ARV Polymeric NP (2023–2024)	Dual/tri-ARV polymer nanocarriers	Multi-drug targeting to reservoirs	Synergistic viral suppression & resistance prevention	[157]
Dendrimers & Polymer Conjugates	Mannose-Functionalized Dendrimers (2024)	Glycosylated dendrimers	Macrophage targeting	Enhanced macrophage uptake & reservoir suppression	[158]
Dendrimers & Polymer Conjugates	Dendrimer-microRNA Nanocomplex (2023)	PAMAM-miRNA dendrimer	miRNA immunomodulation	Improved immune modulation & intracellular transport	[159]
Inorganic Nanoparticles (Gold, Silica)	Gold NP-Enhanced ARV Delivery (2024)	Surface-modified gold NPs	Cytosolic ARV delivery	Increase intracellular accumulation & antiviral effect	[160]
Biomimetic Systems (Exosomes, Cell-mimicking NPs)	Exosome-Mimetic Liposomes (2023)	Exosome-like liposomal siRNA	Endosomal escape & cytosolic siRNA	Superior siRNA delivery vs. traditional liposomes	[159]
Gene / RNA Nanocarriers (CRISPR, RNAi, mRNA)	mRNA-LNP for Latency Reversal	LNP delivering Tat mRNA	Latent HIV reactivation	Robust latency reversal in primary T-cells	[161]
CRISPR/Cas9 Ablation	CRISPR-RNP Vesicle Therapy	Vesicle-CRISPR RNPs	HIV proviral genome knockout	Eliminated HIV proviral DNA in vitro	[162]

The table present a comprehensive set of recent, real-world case studies has been incorporated across all nanoparticle classes to strengthen translational relevance. These studies highlight key advancements including > 50-fold drug accumulation in lymph-node tissues in primate models, 189-fold improvement in bioavailability via vesicular systems, ~ 6-fold enhancement in oral delivery with PLGA nanoparticles, macrophage-targeted dendrimer platforms, enhanced intracellular uptake using gold nanoparticles, and mRNA-lipid nanoparticles capable of reversing HIV latency ex vivo

## Future directions and research priorities

### Nanoparticle-based delivery of RNA therapies for HIV treatment

RNA-based therapeutics, including RNA interference (RNAi) and messenger RNA (mRNA) vaccines are been explored as treatment and prophylaxis. They are used enhance stability, membrane penetration, and controlled intracellular release. RNAi strategies, particularly small interfering RNAs (siRNAs), can silence essential viral genes or host dependency factors [126]. Nanoparticles such as cationic liposomes and polymer-based carriers have been designed to deliver siRNA to HIV-infected cells. Liposomes encapsulating siRNA against the HIV *gag* gene demonstrated potent antiviral activity even in drug-resistant viral strains. Cationic polymeric nanoparticles further ensure endosomal escape, allowing siRNA to reach the cytosol and exert gene-silencing effects. mRNA-based vaccines have proven to be effective with preclinical studies showing that lipid nanoparticles (LNPs) loaded with mRNA encoding the HIV *gp120* envelope protein. This has been reported to robust CD4⁺ and CD8⁺ T-cell responses and neutralizing antibodies [127] against HIV infection. LNPs stabilize mRNA and facilitate translation within antigen-presenting cells, leading to enhanced immunogenicity. Mucosal mRNA-NP platforms are under investigation to generate localized immune responses at sites of viral entry, providing a new avenue for preventive vaccination [128]. Recent advances have introduced self-amplifying RNA (saRNA) nanoplatforms as a next-generation approach for HIV vaccination. Unlike conventional mRNA, saRNA encodes both the antigen and a replicase enzyme, allowing intracellular RNA amplification and prolonged antigen expression at significantly lower doses. When formulated within lipid nanoparticle carriers, saRNA constructs have demonstrated enhanced immunogenicity, durable CD4⁺ and CD8⁺ T-cell responses, and potent neutralizing antibody production in preclinical HIV models. Moreover, saRNA-LNP systems enable dose-sparing, reduced manufacturing cost, and sustained antigen availability [129].

### Nanoparticle-Based delivery of Gene-Editing technologies (such as CRISPR)

**Fig. 4:**
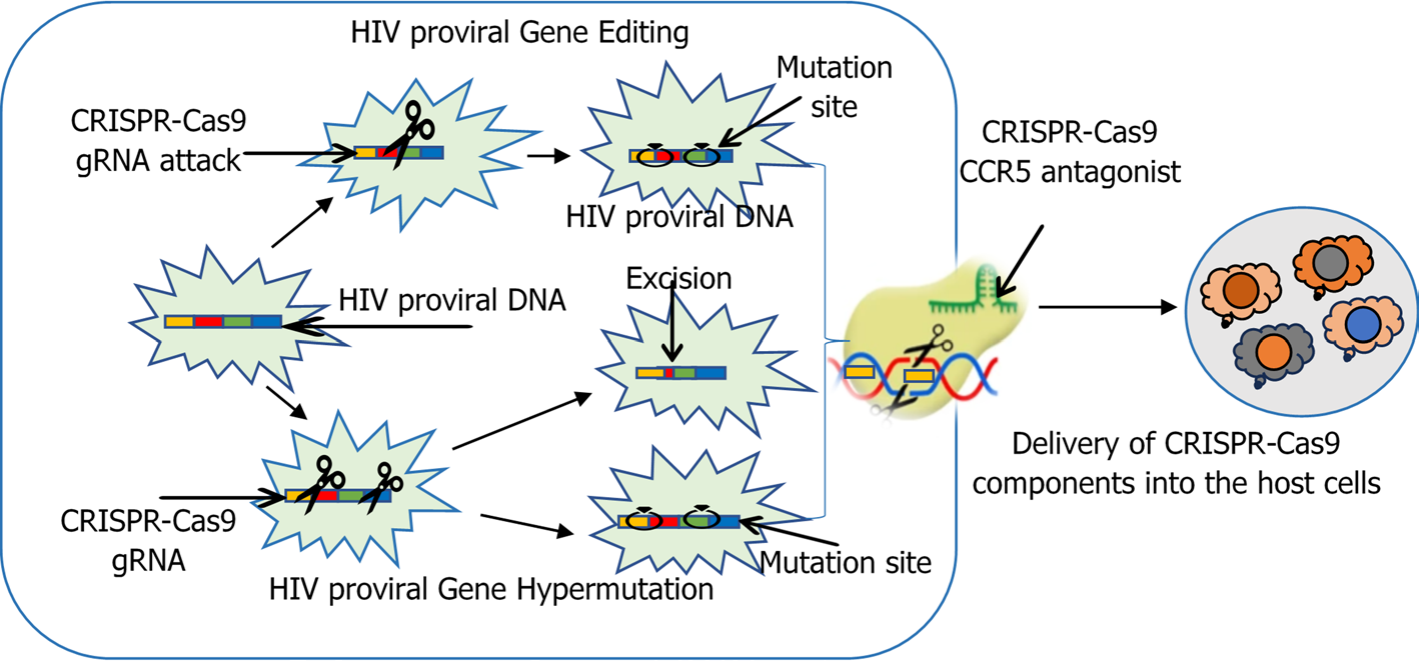
CRISPR-Cas9 and gene-editing technology in treating HIV by targeting and removing integrated viral DNA from host cells. The figure illustrates different mechanisms by which CRISPR–Cas systems can target and cleave integrated HIV proviral DNA. Guide RNAs direct Cas enzymes to specific viral sequences within the host genome, enabling precise excision or disruption of HIV genes. Delivery of CRISPR–Cas components can be achieved using nanoparticle or viral vector systems, leading to editing events within infected immune cells

CRISPR-Cas9 and other gene-editing technologies have been considered in treating HIV by targeting and removing integrated viral DNA from host cells. Nanoparticles are required to transport these sensitive biomolecules effectively and safely [130]. The gene-editing technologies (such as CRISPR) include the delivery of CRISPR-Cas9 components, where nanoparticles transport the CRISPR-Cas9 system (Cas9 protein and guide RNA) to infected cells while shielding the components from nuclease degradation. In animal models, lipid nanoparticles (LNPs) carrying CRISPR-Cas9 targeting the HIV long terminal repeat (LTR) regions efficiently removed proviral DNA, as depicted in Fig. 4 [131]. Functionalized nanoparticles to target infected cells is another technology whereby nanoparticles are allowed to selectively distribute CRISPR components to HIV-infected cells, minimising off-target effects. Antibody-coupled Nanoparticles (LNPs) coupled with anti-CD4 antibodies successfully delivered CRISPR-Cas9 to CD4 + T cells, the principal targets of HIV infection [132]. Nanoparticles can administer CRISPR-Cas9 alongside immune-stimulating drugs or ARVs to eliminate latent virus while suppressing active replication. Gold nanoparticles (AuNPs) are usually functionalized with both CRISPR components and interleukin-15 (IL-15), resulting in viral DNA excision and increased immune activation. Gene-editing systems offer a direct solution to the challenge of latent HIV reservoirs by enabling the excision of integrated proviral DNA from host cells where ART remains ineffective [133]. Furthermore, emerging strategies are expanding the scope of CRISPR delivery. Exosome-based delivery systems are being design as natural nanocarriers due to their intrinsic immune compatibility, stability in circulation, and tropism for T cells. Similarly, CRISPR-loaded smart nanoparticles are being developed to co-deliver antiretrovirals or immunomodulators alongside gene-editing tools, enabling simultaneous suppression of viral replication and clearance of latent provirus. minimizing off-target effects. Additionally, emerging base and prime editing nanoplatforms are being explored as safer genome-correction strategies for HIV. Base editors enable precise single-nucleotide conversion without generating double-strand breaks, reducing risks of insertion deletion mutations. Prime editors extend this capability by installing small insertions, deletions, or base substitutions using a reverse-transcriptase-guided mechanism, offering greater editing versatility [134].

### Smart Stimuli-Responsive nanocarriers

Stimuli-responsive nanocarriers are engineered to release antiretroviral drugs in response to specific biological cues such as pH shifts, enzymatic activity, or redox conditions. This enables localised and sustained drug action at sites of viral persistence. These smart delivery systems enhance drug penetration into hard-to-reach anatomical reservoirs, ensuring targeted delivery where the virus often evades conventional therapies. This system improves drug concentration and activity at the site of infection, minimising toxicity and the risk of resistance. This dual advantage addresses two of the most enduring challenges in ART pharmacology, such as inadequate reservoir targeting and systemic side effects [135].

### AI and machine learning in nanoparticle optimisation

Artificial intelligence (AI) and machine learning (ML) are recognised an important tools for advancing the design and clinical translation of nanoparticle-based HIV therapeutics. AI-driven algorithms can predict the optimal nanoparticle properties such as particle size, surface charge, ligand density, and drug loading efficiency. For example, predictive ML models have been used to design lipid nanoparticles (LNPs) with improved stability and enhanced delivery efficiency for RNA-based therapies [136]. AI can also personalise therapy. Patient-specific pharmacokinetic and pharmacogenomic data can be integrated into predictive models to forecast drug distribution, clearance rates, and likely toxicity. This enables precision tailoring of nanoparticle formulations to reduce inter-individual variability and improve safety-efficacy balance. Moreover, AI-enabled digital adherence monitoring systems such as ingestible sensors, smartphone-linked nanocarriers, and smart wearables can track patient compliance in real time. When coupled with algorithm-based dose adjustment systems, these innovations may proactively prevent viral rebound and resistance [137]. AI-based simulations can also promote regulatory approval by reducing the reliance on lengthy in vivo testing. Virtual screening of nanoparticle libraries, coupled with computational modelling of biodistribution and immune interactions, offers a cost-effective pathway to identify the most promising candidates before clinical trials [138]. Recent advances in AI are accelerating nanoparticle design through multi-objective optimization (MOO) frameworks that simultaneously evaluate stability, drug loading, release kinetics, and biocompatibility. Machine-learning models integrated with MOO enable rapid screening of formulation variables and prediction of optimal nanocarrier compositions. Additionally, AI-based in-vitro–in-vivo correlation (IVIVC) tools are being applied to forecast clinical performance from laboratory data, reducing experimental workload and improving translational efficiency [139].

### Long-Acting and personalised nanomedicine for HIV treatment and prevention

Long-acting nanoparticle formulations represent an important of strategies to improve adherence and reduce the burden of daily ART. Injectable lipid-based nanoparticles and polymeric systems have already demonstrated the capacity to maintain therapeutic plasma drug concentrations for weeks to months, thereby minimising adherence-related failures [140]. The FDA-approved long-acting injectable combination of cabotegravir and rilpivirine exemplifies this approach, and ongoing research into nanoparticle-based biodegradable implants and hydrogel depots promises even longer dosing intervals with fewer clinic visits. In parallel, localised prevention strategies using vaginal and rectal nanoparticle gels or implantable nanocarriers provide site-specific protection where viral transmission occurs. Such platforms reduce systemic toxicity and empower users by offering discreet, user-controlled prevention methods, particularly important in communities where stigma or gender dynamics hinder adherence to daily oral PrEP [141]. Polylysine–heparin functionalized solid lipid nanoparticles (fSLNs) were engineered as a vaginal microbicide delivery system for HIV prevention, using tenofovir as the model drug. The nanoparticles were prepared via a modified phase-inversion and layer-by-layer method, with Box–Behnken design assessing formulation variables. Characterization showed platelet-like particles with a mean diameter of 153 nm, zeta potential of − 51 mV, and 8.3% encapsulation efficiency. The fSLNs were non-cytotoxic to vaginal epithelial cells and predicted to enhance cellular uptake of hydrophobic microbicides, offering promise for effective topical HIV prevention [142]. Additionally, digital-twin pharmacokinetic modeling used to simulate nanoparticle biodistribution and predict optimal clinical dosing. By integrating patient-specific physiological data with mechanistic PK/PD models, digital twins enable real-time prediction of nanoparticle circulation, tissue accumulation, and clearance. This approach supports individualized dosing strategies, reduces reliance on animal testing, and enhances translation of nanotherapeutics from bench to bedside [143].

### Regulatory evaluation

**Fig. 5:**
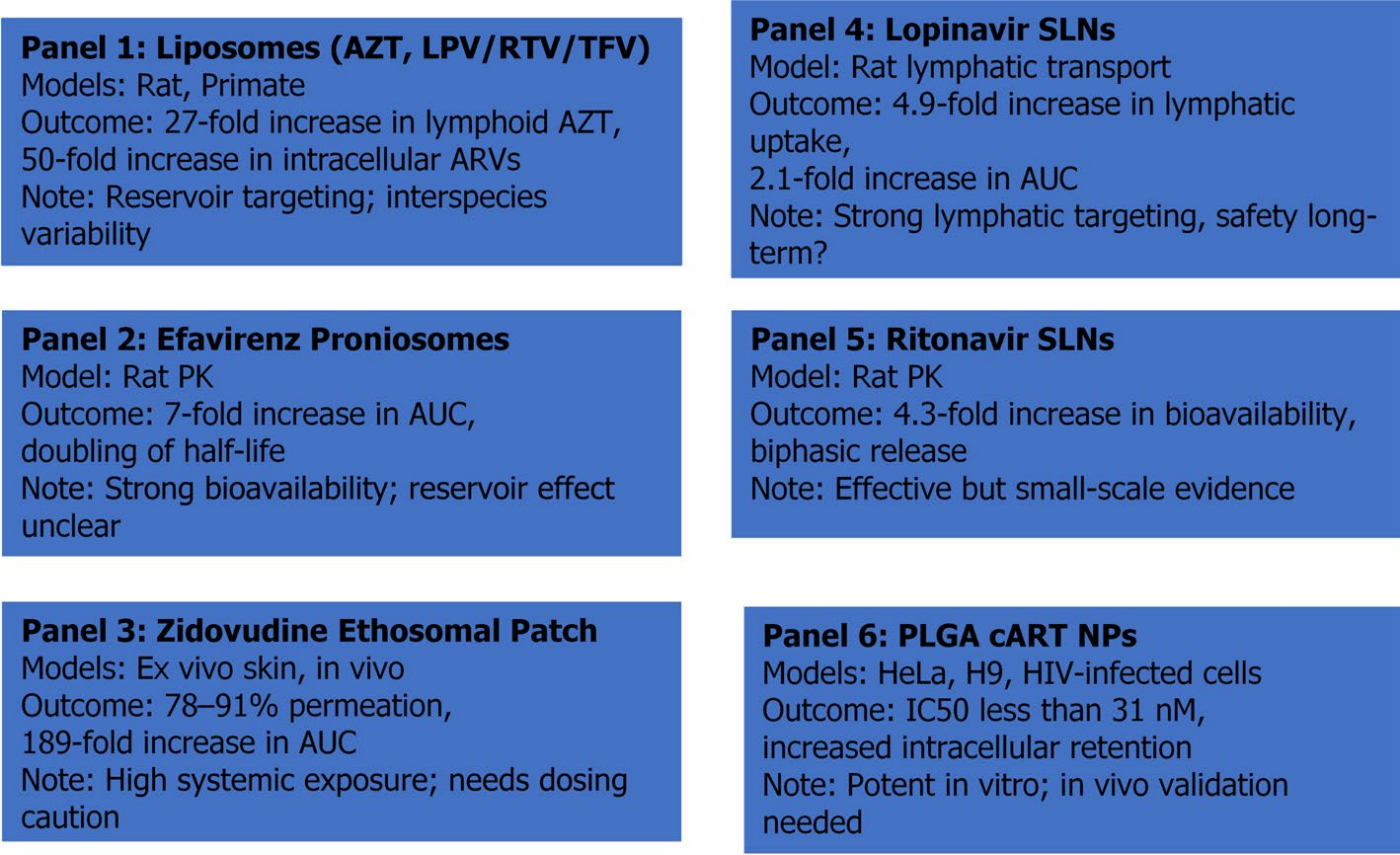
Preclinical case studies of nanoparticle-based antiretroviral systems. Liposomal zidovudine and lopinavir/ritonavir/tenofovir showed up to 27- and 50-fold increases in reservoir uptake. Efavirenz proniosomes improved oral exposure seven-fold and doubled half-life, while zidovudine ethosomal patches enhanced permeation and raised bioavailability nearly 189-fold. Lopinavir and ritonavir SLNs increased bioavailability 2–4 fold with sustained release. PLGA cART nanoparticles achieved efficient intracellular retention and potent HIV-1 inhibition (IC50 < 31 nM). Together, these systems demonstrate improved bioavailability, reservoir targeting, and antiviral potency, though broader in vivo validation remains essential

Regulatory evaluation of nano-formulations for HIV therapy requires consideration of nanoparticle-specific attributes beyond conventional drug criteria, including surface chemistry, particle size distribution, zeta-potential, and release kinetics, as these parameters directly influence biodistribution and long-acting pharmacokinetics. Agencies such as the FDA and EMA now apply nanotechnology guidance that emphasize detailed physicochemical characterization, stability profiling, immunotoxicity assessment, and biodistribution studies, particularly for long-acting injectable systems like cabotegravir and rilpivirine [144]. These products must demonstrate consistent nanoparticle quality, controlled release behaviours, and absence of unexpected tissue accumulation or immune activation. The requirement for extended non-clinical safety studies, real-time stability data, and post-marketing surveillance reflects the evolving regulatory emphasis on long-term safety and manufacturing reproducibility in HIV nanomedicine development (Fig. 5).

### Integration of digital health and smart drug delivery

The combination of digital health technologies with smart drug delivery systems holds a transformative feature in personalized medicine. Wearable biosensors and mobile health (mHealth) applications enable continuous, real-time tracking of drug concentrations and immune system markers [145]. This real-time feedback can autonomously trigger nanoparticle-mediated drug release in response to specific biological cues, such as viral load fluctuations, ensuring timely and tailored therapeutic interventions. The integration of these digital tools into nanomedicine platforms overcomes behavioral and systemic barriers such as poor adherence, delayed dosing, and toxicity. Moreover, adaptive dosing algorithms and behavioral feedback loops foster a dynamic treatment paradigm, transforming static, one-size-fits-all approaches into responsive, patient-centered care models [146].

## Conclusion

Nanoparticle-mediated drug delivery systems have emerged as a transformative advancement in HIV therapeutics, addressing persistent limitations of conventional antiretroviral therapy (ART). Platforms including liposomes, polymeric nanoparticles, dendrimers, nanocrystals, and lipid-based carriers have demonstrated the ability to improve pharmacokinetics, enable controlled release, and enhance drug distribution to sanctuary sites, ultimately improving adherence and therapeutic outcomes. Looking forward, alignment with global nanomedicine policy frameworks from the WHO, NIH, and EMA will be essential to support safe and equitable translation of HIV nanotherapeutics. Genome editing platforms, such as CRISPR-Cas9 delivered via nanoparticles, hold potential for directly excising or silencing latent proviral DNA, moving HIV treatment beyond viral suppression toward functional cure strategies. Multifunctional nanocarriers capable of co-delivering antiretrovirals, latency-reversing agents, and immunomodulators could simultaneously suppress replication, expose hidden reservoirs, and enhance immune clearance. In parallel, the integration of nanotechnology with HIV vaccines and RNA-based platforms may enable durable immune priming and synergistic therapeutic effects. Artificial intelligence driven optimization of nanocarrier design will further refine targeting specificity and safety. Despite these advances, challenges remain, including nanotoxicity, manufacturing scalability, regulatory adaptation, and affordability. Overcoming these barriers through proper safety testing, innovative large-scale production, and equitable pricing models will be essential to ensure global accessibility.

## Data Availability

No datasets were generated or analysed during the current study.

## Abbreviations

AIDS: Acquired immunodeficiency syndrome
ART: Antiretroviral therapy
ARV: Antiretroviral
AuNPs: Gold nanoparticles
BBB: Blood–brain barrier
CD4+ T cells: Cluster of differentiation 4T cells
CNS: Central nervous system
CpG DNA: Cytosine–phosphate–guanine DNA
CRISPR: Clustered Regularly Interspaced Short Palindromic Repeats
CTL: Cytotoxic T lymphocyte
CXCR4: C–X–C motif chemokine receptor 4
DNA: Deoxyribonucleic acid
EPR: Enhanced permeability and retention effect
EMA: European Medicines Agency
FDA: Food and Drug Administration
GSH: Glutathione
HAND: HIV-Associated Neurocognitive Disorders
HIV: Human immunodeficiency virus
IC50: Half maximal inhibitory concentration
IL-15: Interleukin-15
IM: Intramuscular
IV: Intravenous
LNPs: Lipid nanoparticles
LTR: Long terminal repeat
mHealth: Mobile health
mRNA: Messenger ribonucleic acid
MPS: Mononuclear phagocyte system
NRTIs: Nucleoside reverse transcriptase inhibitors
NNRTIs: Non-nucleoside reverse transcriptase inhibitors
NPs: Nanoparticles
PAMAM: Poly(amidoamine)
PBPK: Physiologically based pharmacokinetics
PEG: Polyethylene glycol
PEGylation: Polyethylene glycol modification
PIs: Protease inhibitors
PLGA: Poly(lactic-co-glycolic acid)
PrEP: Pre-exposure prophylaxis
RES: Reticuloendothelial system
RNA: Ribonucleic acid
RNAi: RNA interference
ROS: Reactive oxygen species
siRNA: Small interfering RNA
SLNs: Solid lipid nanoparticles
TLR: Toll-like receptor
TNF: α-Tumor necrosis factor-alpha
VLP: Virus-like particles
WHO: World Health Organization
ZnO NPs: Zinc oxide nanoparticles
